# Medical News and Miscellany

**Published:** 1887-04

**Authors:** 


### Medical News and Miscellany.

Surgery in Texas.—"We have received the report of the Special Committee on Surgery, appointed by the State Medical Society of Texas, Dr. George Cupples being chairman and reporter. It makes a quarto of seventy-two pages and embraces the tabulated statistics regarding 4,293 operations performed by Texas surgeons. The report is a valuable one; it represents an immense amount of intelligent labor, and cannot fail to be a stimulus and benefit to the very wide-awake practitioners of the Lone Star State. The statistics show good, and even brilliant results in most lines of surgery. In amputations of the thigh the number of cases is 132, with 50 deaths; those of the leg are 166, with 27 deaths; those of the arm, 65, with 60 deaths; and those of the forearm, 66, with 2 deaths. These figures are even better than those, for example, of Max Schede, in which the operations are done with every possible antiseptic precaution. It must be remembered, however, that as the statistics collected by the Texas Committee are voluntarily contributed, it is quite possible that all the bad cases are not reported. The report shows that the Texas surgeon is quite able to cope with all varieties of surgery, or even the most difficult.

*New York Medical Record.*

The *Record* makes an error in the figures, which, in justice to all, we correct. In amputations of the thigh, including 8 of hip-joint, the number should be 122, (and not 132), with 50 deaths; of the leg 166, with 22 deaths instead of 27; of the arm 75, instead of 65, with 11 deaths instead of 66, as reported by the *Record.*

---

TEXAS STATE MEDICAL ASSOCIATION.—Report of the Special Committee on Surgery, presented at the annual meeting, at Dallas, April 27th, 1886. Committee, E. J. Beale, M. D., A. G. Clopton, M. D., B. F. Eads, M. D., J. M. Pace, M. D., D. F. Stuart, M. D., George Cupples, M. D., Chairman and Reporter. Folio pp. 76. Austin, Texas.

One may truly say that these are the most valuable statistics given us since the publication of the *Medical and Surgical History of the Civil War.* It is but the nucleus, as the compiler tells us, of what shall follow. Heretofore those who sought surgical statistics from private note would necessarily need scour the journals for published cases, and generally abandoned the effort, not getting material enough for a compilation, but this is one State in the Union which can boast of work better performed, of results more satisfactory, of methods more in harmony with the existing knowledge of surgical science. There may be, others, but if there are they have failed to give it to us and the rest of the profession in the manner of our Southern confréres.

To the gentlemen then of the committee, to Dr. Cupples in particular, and the men whose work is herein gathered, the profession at large owe a debt which could in no better way be cancelled than by following the path in which they have taken the lead. The example is a *noble one*; let us of the North take precedent by it, and enrich medical literature by the most valuable aid which it is possible for it to receive.

*Med. Register, Pa.*

---

The following handsome compliment from an editorial in the *New Orleans Medical and Surgical Journal* is highly appreciated, especially as the example of Texas is cited as worthy of emulation:

"Texas is an empire; the distances to be crossed by the majority of men making towards a given point within her boundaries, are immense, and yet the ateendants at the annual meetings of her Medical Association are numbered by hundreds; we would boast a throng, if the number touched one hundred. The volume of Transactions, issued by the Texas State Medical Association, last year, has been hailed everywhere as a work of merit; a real contribution to the literature of our art. * * * Have our readers seen the Report of the Committee on Surgery of the Texas Association, issued in a separate volume as an appendix to the fine volume of Transactions already mentioned? If not, they have been spared a jealous mortification to which we own ourselves no strangers. It is a work for a whole State to be proud of, and contains, possibly, more meat than any publication ever put forth by a body of Southern professional men."

---

Many physicians prefer Cincho Quinine to the sulphate of quinine in intermittents, especially with children on account of its tastelessness.

---

CAPITAL PUNISHMENT BY ELECTRICITY.—A legislative commission of medical men has been appointed in New York, to inquire into the merits of various devices for putting murderers to death, with less repulsiveness than by the method now practiced—hanging, which, in most cases, means strangling; and is understood that they will shortly report to the State Legislature in favor of the adoption of electricity. It is thought a bill will be passed abolishing execution by the gallows, and substituting electricity. This would, indeed, be a reform much needed.

We are opposed to capital punishment. Statistics show that in those States where hanging is practiced, crime is on the increase; hence the ostensible object, and only excuse are defeated. We doubt the right of a State to commit murder—for such is hanging—or any other method of execution. We are also opposed to it on economic grounds. It would seem that one object, at least, of capital punishment is, to rid society of those who are dangerous to its peace. This can be as effectually accomplished by imprisonment and labor. Let a man who has forfeited his life according to our laws—not be executed, but put to work as a producer, and made to contribute to the wealth of the State. As each adult male is estimated to be worth about $800 a year as a producer, the State is loser to that amount every time a man is hanged, and as shown above—no good is accomplished. As to the mere method of murdering a criminal, we suppose it makes but littie difference with him, whether by rope, axe or electricity; it must be horrible to contemplate. Sullivan (in Mikado) thus facetiously describes it:

"Sitting in solemn silence in a dull, dark dock—
In a pestilential prison with a life-long lock,
Awaiting the sensation of a short, sharp shock
From a cheap and chippy chopper on a big black block."

*Can* anything be more horrible? Away with "judicial murder."

VALUABLE PAPERS.—We call attention to the unusually large number of valuable papers in this issue of the JOURNAL. It is, indeed a "star" edition, every copy is worthy of careful preservation.

It is believed that Dr. Cupples' case—resection of intestine for g. s. wound of the bowel is the first case south of the Potomac. A singular coincidence is, that his case and Dr. Kingsley's were done the same day—Sunday. It is quite an event that two laparatomies, one for pyosalpinx and ovarian cyst, the other for g. s. wound, should occur on one day in a Texas town.

In this connection we will remark that in examining the Report on Surgery, European Surgeons noticed, and were struck with the fact that so many of the major operations were performed without antiseptic measures, and without the facilities now afforded in every hospital. Undêr those circumstances the results are wonderful. Those gentlemen also observed and commented on the fact that Texas Surgeons show such readiness of adaptation to circumstances, many operations being unique and original. The condition of our West Texas atmosphere accounts for the former, and *necessity*, for the latter, as we once said, when a country Doctor is in a tight place he has to "think it out"; he has no time to consult books, and is out of reach of consultation; and in emergencies he "thinks it out," with the celerity of instinct! and saves his patient!

We sincerely trust the publication of these papers, and a knowledge that the world is watching our Surgeons with interest, and applauding their skill, will induce operators to send us their cases. The JOURNAL is the door to the text-books, and to—fame! Use it.

---

HONORS TO PROF. DUDLEY S. REYNOLDS, OF THE "BOUNDLESS WEST."

At the Sixth Annual Commencement of the Medico-Chirurgical College, Philadelphia, held on 7th inst.; the degree of F. M. C. R. was conferred upon Prof. Dudley S. Reynolds, the able and progressive editor of "Progress," published at Louisville. Prof. Reynolds delivered an address, by invitation, before the faculty and graduating class, taking for his subject " A History of the Growth ("Progress") of Medicine," and after the exercises a banquet was given in his honor. Prof. Pancoast presided, and the toast, "The Boundless West," was responded to by Professor R. in his usual happy style. There could have been no more fitting selection for the response to this toast than this distinguished representative of that glorious country "on which the sun never sets," the "Boundless West."

---

THE VERY LATEST.—The committee of arrangements have perfected a contract with the Musical Union to have the Grand Opera of Balshazzar on Tuesday evening, April 26, the first night of the Medical Convention; benefit of the Tenor, Mr. Durham, an Austin amateur. Delegates and their ladies will be complimented with free tickets. It is contemplated to give magic lantern exhibitions of pathological specimens on the evening of the President's address, Thursday, in the same hall. The management is under the Texas Miscroscopic Association, of which Dr. McLaughlin is President. Those who fail to come will regret it.

---

EXHIBITS AT THE TEXAS MEDICAL CONVENTION.—The Secretary has been for some time in correspondence with manufacturers of Pharmaceuticals, Instruments and Surgical Appliances with regard to the April meeting of the State Medical Association, and we are authorized to state that the following firms will be represented by agents, with full lines for exhibition, (and samples for distribution). The indications are, that this feature of the meeting will be unusually attractive:

Messrs. Sharpe & Dohme..................Baltimore.
" Parke Davis & Co.,..............Detroit.
" W. R. Warner & Co.,.............Philadelphia.
" Thomas Leming & Co., (Nestles Food), New York.
" Reed & Carnrick,.................New York.
" J. P. Bush Manufacturing Co.,....New York.
" J. W. Lambert, (Listerine),........St. Louis.
" Seaburry & Johnson, ............New York.
" Allison & Johnson, (Clark Chair), Indianapolis.
" Geddes M'f'g. Co., Ext. Hemlock, [London, W. C. and Boston.*
" R. A. Robinson & Co.,..........Louisville. *

*[By sample only, no agent: The Hemlock is for distribution to those who will agree to use it in practice-]

---

The name of our sprightly contemporary at Waco is changed from the "Texas Druggist" to the "Texas and Southwestern Druggist." The Druggist is doing yeoman service in the cause of "higher Pharmacy" and organization.

---

The Texas State Pharmaceutical Association will meet in Fort Worth the Second Tuesday, 10th day of May, prox. *The Texas S. W. Druggist* offers a cash prize of $50 for the best essay on any subject connected with Pharmacy presented before the State Pharmaceutical Association at this meeting.

---

The Central Texas Medical Association held its third quarterly meeting in Waco, Tuesday, April 12th inst. We have had no report to date, but understand the Association is in a flourishing condition.

---

Papers for the Sections.—The following is a list of papers to be read at the coming meeting of the The Texas State Medical Association, as far as reported to the Secretary, up to the time we go to press: J. W. McLaughlin, M. D., Chairman, Section on Obstetrics and Diseases of Women. 1. Management of the Third Stage of Labor, from the Standpoint of Personal Experience," by S. H. Stout, M. D., Cisco, Texas.

Section on Ppactice Medicine, Etc., J. F. Y. Paine, M. D., Chairman:

1. Mental Alienation a Sequel of Intermittent Fever: E. Goldman, M. D., Galveston.
2. Babies and Their Troubles, by C. L. Gwyn, M. D., Galveston. [Secretary of Section].
3. Spinal Irritation, by J. W. Carhart, M. D., Lampasas.
4. Typho Malarial Fever, by E. J. Ward, M. D., Waxahachie.
5. Pneumonia, by J. C. Milner, M. D., Comanche,

Section on Surgery and Anatomy. J. D. Osborn, M. D., Chairman. Treatment of Internal Hemorrhoids, by Hypodermic Injection, by Q. A. Shuford, M. D., Tyler.

Section on Electro Therapeutics: F. T. Paine, M. D., Chairman. 1. Three years work with electricity, by F. T. Paine, Comanche.

Section on Ophthalmology and Otology, T. J. Tyner, M. D., Chairman.

1. Report on Ophthalmology and Otology, T. J. Tyner, M. D., Chairman.

2. Iritis, by R. H. Chilton, M. D., Dallas.

3. The Syringe in Ear Diseases, by J. R. Briggs, M. D., Dallas.

4. A Case of Neuro Retinitis—total loss of vision in both eyes, followed by complete restoration in right eye, and a partial one in the left, by J. H. Smith, M. D., Dallas. [Secretary to the Section].

"WHAT IS A BANQUET?" Our distinguished contemporary of the *Journal American Medical Association* takes us to task for saying "a banquet without wine would be a hollow mockery." Custom has so made it, at least,—in modern times;—and so general is the flow of wine at all convivial gatherings, that its absence is like taking Hamlet out of Hamlet. Prof. Davis is such a teetotaler, that he seems to regard "a wine supper and a debauch," as synonymous; we do not, by any means, and we were quite explicit in the statement that fear of indiscretion on the part of our guests did not enter at all, into the considerations which induced the committee to dispense with both, wine and banquet.

---

TO MEMBERS OF THE TEXAS STATE AND MEDICAL ASSOCIATION.—A copy of the JOURNAL has been mailed to every member of the Association, whose name appears on the roll, whether a subscriber or not. To those who *are not* subscribers it is sent, with the editor's compliments, as a *souvenir* of the Austin meeting. An extra edition has been printed, and it is intended as a *memorial* to our deceased brethren, and commemorative of the Austin meeting.

---

TO WHOM IT MAY CONCERN: Of the number who joined at Dallas, the JOURNAL has been regularly sent for one year, to twenty-five. True, it was not authorized, but, as it was, in no instance returned, and no request made, to discontinue it, those twenty-five are, of course, considered subscribers; and this notice is intended as a gentle reminder that the coming meeting will be a very appropriate time to settle,—and that it takes money to publish a monthly Medical Journal.

---

A DESERVED COMPLIMENT.—*Progress*, (Louisville, Ky.) in a lengthy review of the transactions of the Texas State Medical Association for '86, in which the palm for excellence is yielded to Texas, says of Dr. Becton's address :

"The annual address of the President, Dr. E. P. Becton, was so much above the average of its kind as to have attracted wide attention and is still being discussed in the medical journals. Some have considered it rather over-wrought in sentiment and fervid in style. We do not consider questions of style and taste in a composition of this kind, so long as they are not bad. It was intended to fire the ambition and renew the mutual pledges of fraternity from the standpoint of a zealous churchman. To justify the standard of his own address, we must accord to Dr. Becton a distinguished place as a citizen and a member of the medical profession."

---

PERSONAL.—We regret to announce that our distinguished President, Dr. T. H. Nott, is suffering from serious impairment of his general health, from want of the necessary rest and recreation, incident to a large and laborious practice extending over a vast area of country, and necessitating much riding. He has gone to Waco to recuperate during the brief interval before the meeting; but like the thrifty farmer who wanted the men to "clean up the yard" while they were "resting," the Doctor is at work on his address, and making other active preparations for the meeting. We sincerely trust that his health will permit of his being with us and giving us one of his best efforts.

---

### "A LITTLE PREVIOUS"?

---

DR. F. E. DANIEL, AUSTIN, TEXAS :

*Dear Doctor*—My attention has just been called to a little notice from the Editor of the Kansas City *Medical Index*, on page 99 of March issue, referring to Dr. W. H. Wilkes. The Editor thinks your notice of Dr. Wilkes a little previous, but I am happy to say that it was not much so, for at the last meeting of the Faculty of the Medical Department of the University of Kansas City, Dr. Wilkes was elected to fill the Chair of Obstetrics and Diseases of Children.

I write this in justice to Dr. Wilkes.

Yours sincerely,

JNO. W. JACKSON,

President of Faculty.

The Kansas City *Medical Index*, having challenged our assertion in February number, "as a little previous," that Dr. Wilkes had accepted a professorship in the Kansas City Medical College, we copy the above, which is official. We will state that our authority for saying a professorship had been offered Dr. Wilkes, was beyond question. The only mistake was in our placing Dr. Wilkes in the wrong college—the Kansas City Medical College instead of the Medical Department, University of Kansas City.

---

SEPARATING THE LICENSING FROM THE TEACHING POWER.—We learn from the *N. Y. Medical Record* that Minnesota has passed a law which robs a diploma of its supposed authority. Though the courts of many States have decided that "a diploma conveys no right to practice medicine"—being what Webster defines it—"a record of a degree having been conferred," popular opinion holds it to be all sufficient to entitle the holder to practice anywhere, and custom has sanctioned it. The provisions of the Minnesota law, are: 1. The appointment of a Board of Examiners. 2. No member to serve more than two terms. 3. Two members are to be homœpaths. 4. All persons hereafter commencing the practice of medicine in the State must pass an examination upon the different branches. 5. Before entering upon said examination, the applicant must produce evidence of having attended three courses of lectures, if date of diploma is later than '87. 6. Power is given the board to refuse or revoke license for unprofessional, dishonorable or immoral conduct. 7. No member of any existing medical faculty eligible to appointment on the new board.

The last section divorces entirely the licensing from the teaching power.

---

AUSTIN GRADUATES.—Messrs. Justus Duffau, Frank Litten and Clark Red, of Austin, graduated last month at Jefferson Medical College, Philadelphia. Mr. Duffau who was a student of Dr. Joseph Cummings, took the gold medal for best original paper on obstetrics. The examinations are said to have been unusually strict, as, in response to pressure of public sentiment, the Jefferson Medical College has raised the standard of requirements; and a large number of applicants, were rejected, including, it is reported, the foreman or "president" of the graduating class. Mr. Litten is a son of our esteemed fellow-citizen, Dr. J. M. Litten, an old resident practitioner of Austin, and studied under his instruction. He will remain in hospital at Philadelphia some months to study certain special branches. We have not the pleasure of knowing Dr. Red, but we welcome all these gentlemen to the ranks. Such men are a credit to the profession and to their State.

